# New Plant Breeding Techniques Under Food Security Pressure and Lobbying

**DOI:** 10.3389/fpls.2018.01324

**Published:** 2018-09-19

**Authors:** Qianqian Shao, Maarten Punt, Justus Wesseler

**Affiliations:** ^1^School of Management and Economics, Beijing Institute of Technology, Beijing, China; ^2^Windesheim Honours College, Windesheim University of Applied Sciences, Zwolle, Netherlands; ^3^Department Social Sciences, Wageningen University and Research, Wageningen, Netherlands

**Keywords:** food policy, food security, gene editing, lobbying, political economy

## Abstract

Different countries have different regulations for the approval and cultivation of crops developed by using new plant breeding technologies (NPBTs) such as gene editing. In this paper, we investigate the relationship between global food security and the level of NPBT regulation assuming a World Nation Official (WNO) proposes advice on global NPBT food policies. We show that a stricter NPBT food regulation reduces food security as measured by food availability, access, and utilization. We also find that political rivalry among interest groups worsens the food security status, given the NPBT food technology is more productive and the regulatory policy is influenced by lobbying. When the WNO aims to improve food security and weighs the NPBT food lobby contribution more than the non-NPBT food lobby's in the lobbying game, the total lobbying contributions will be the same for the WNO, and the NPBT food lobby will be more successful in the political process. The NPBT food lobby, however, under food security loses its advantage in the political competition, and this may result in a strict NPBT food policy. Under food security problems implementing stricter NPBT food regulations results in welfare losses. JEL Code: D04, D43, D72, P16

## Introduction

After the 2008 food crisis, the potential fragility of the global food system returned as a major topic in the debate on global food security. Politicians and researchers have suggested several solutions, such as reduction in trade barriers, food aid for food insecure regions, and improving productivity through new agricultural technologies. Modern biotechnology has been considered one of the main contributors to food security (e.g., Ruane and Sonnino, 2011; Sastry et al., 2011; Qaim and Kouser, 2013). However, the importance of the contribution to food security is under debate (e.g., Dibden et al., 2013). Although the topic of this paper is new plant breeding technologies (NPBTs), at several places we refer to experiences gained from the regulation of genetically modified organisms (GMOs) as they bear a number of similarities with NPBTs from a political economy perspective.

The debate about GMOs illustrates that the application of modern biotechnology is not just a scientific problem, but equally a political one involving several interest groups (Miller and Conko, 2004; Graff et al., 2009; Qaim, 2009; Freedman, 2013; Herring and Paarlberg, 2016). This applied to previous technologies, but also applies to NPBTs (e.g., Sprink et al., 2016).

Biotechnology scientists and companies emphasize higher yields and environmental benefits of NPBTs. Opposing organizations, such as Greenpeace and Friends of the Earth, emphasize the potential human health and environmental risks (Rausser et al., 2015; Clancy, 2017), even though there is currently no evidence that proves that NPBTs pose higher risks to either human health or the environment and that rather the opposite can be expected.

International organizations are also involved in the debate. For example, the State of Food and Agriculture report of 2003 on “Agricultural Biotechnology: Meeting the Needs of the Poor?” by the FAO (Food and Agriculture Organization of the United Nations) has been heavily criticized for its “pro-GM” view. Similarly, the report of 2009 on the International Assessment of Agricultural Knowledge, Science and Technology for Development (IAASTD) has been criticized for not paying enough attention to the possibilities of modern biotechnology to address food security: “*But, partly due to the way in which the authors were selected and the main reports were translated into the summaries, the overall message which emerged from the IAASTD was a more restrictive, exclusionary message with an undercurrent against new technology, GMOs, and input-intensive agriculture*.”(McIntyre et al., 2009, p. 38).

In a similar vein, Urs Niggli, Director of the Swiss Research Institute of Organic Agriculture (FiBL), was heavily criticized for his statement NPBTs offer a great potential for organic agriculture (Maurin, 2016). The outcome of the debate on NPBTs, whatever it is, can be expected to affect food policies and therefore, food security.

**Figure 1:**
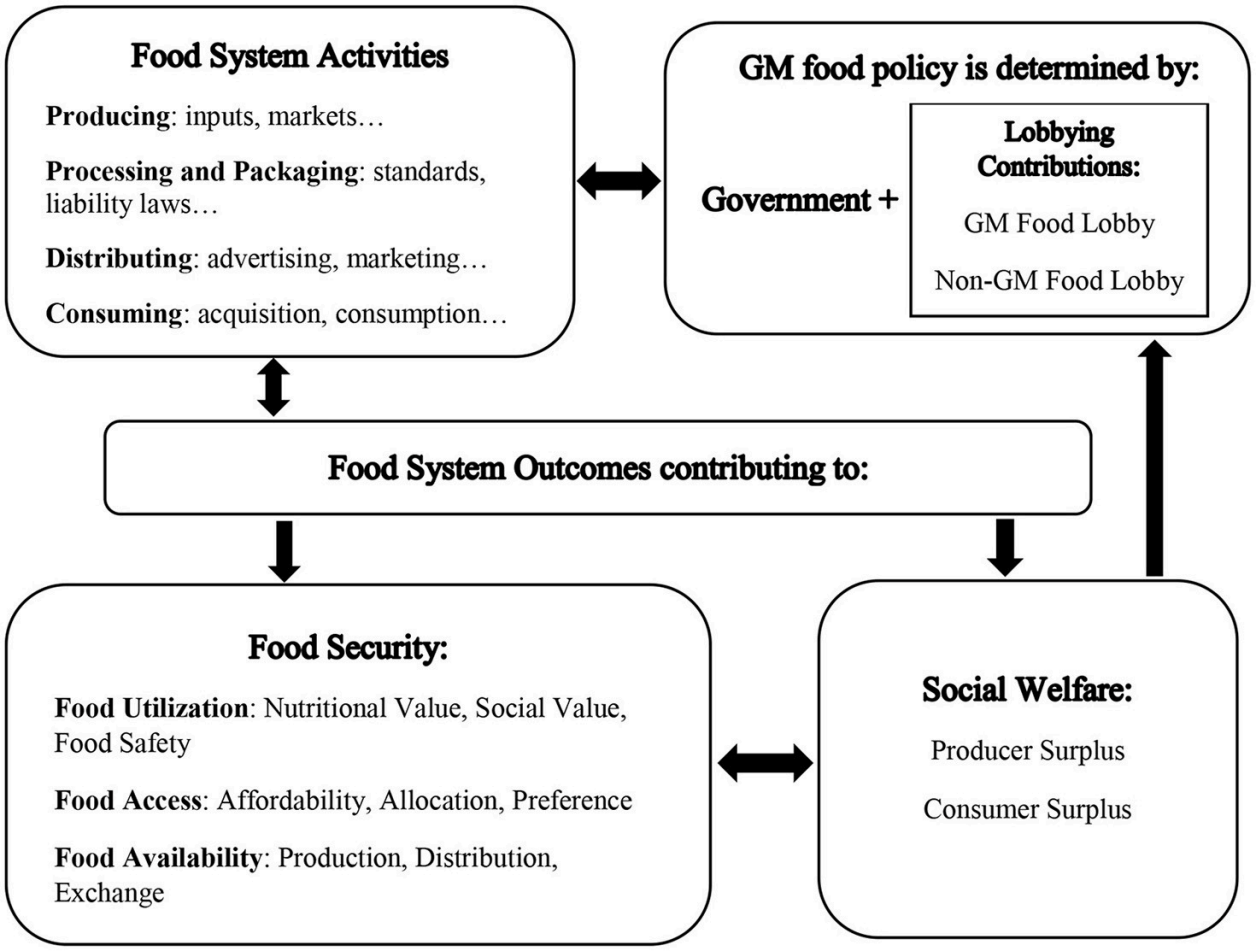
Politically influenced main food system (modified from Ericksen et al., 2009).

In Figure 1, the importance of food policies is illustrated in the food system framework (Modified from Ericksen et al., 2009, p. 28). The NPBT food policy if regulated similar to GMOs will influence the whole food system through production and consumption decisions and will finally result in the changing of prices. The effect will trickle down, affecting food system outcomes. For example, farmers have to comply with food regulations and their labeling standards (Gruère et al., 2009) and coexistence rules (Wesseler and Punt, 2016), seed companies with environmental and food safety regulations (Smart et al., 2017), and countries with international trade agreements (Punt and Wesseler, 2016). In addition, consumers' preferences toward NPBT and non-NPBT food products are influenced by labels and advertisements on food products, media reports, and more (Lusk et al., 2014). Food regulations can also influence the acquisition of food products in the market by implementing stringent or lenient sanitary and phytosanitary standards for food imports. All these policies influence the food system outcomes with impacts on food security and social welfare.

Political differences in the use of NPBTs widen the productivity gap between developing and developed countries by setting barriers on the application of new agricultural technology. As Shiferaw et al. (2011) argue the “hard technology” of genetic modification alone is not enough to improve food security. It needs to be complemented with the “soft technologies” of the development of an appropriate food policy and the establishment of proper institutions that ensure that smallholder farmers can use the technology and profit from it. Biotechnology policy not only influences social welfare directly, but also generates environmental benefits and costs. Many positive environmental effects from GM crops have been observed, such as a reduction in pressure on habitats and biodiversity through increased productivity (Wesseler et al., 2011). Growing GM crops is also less harmful to the environment and human health (Bennett et al., 2004). Similar effects are expected for crops derived from NPBTs.

Several authors applied the political economy theory to study policies on agricultural biotechnology (e.g., Graff et al., 2009; Wesseler and Zilberman, 2014; Tosun and Schaub, 2017; Wesseler et al., 2017). Apel (2010) claims that there are substantial policy and financial benefits that GM food opponents gain from their opposition to GM food technology, i.e., donations, membership fees, and nationally funded policy programs. Some donors provide financial support to NGOs that campaign against GMOs and NPBTs in developing countries (Paarlberg and Pray, 2007). At the same time, the GM food R&D institutes and some seed companies lobby for less strict regulations of biotechnology across countries. The strict GM food regulation in the EU is regarded as a lobbying success of anti-GM food lobby groups (Graff et al., 2009; Qaim, 2009). These conflicting public attitudes and interests in biotechnology manifest in the GM food policy of each country. Therefore, a political economy analysis can offer important insights into the policy formation (Josling et al., 2004).

In this paper, we discuss NPBTs food policies that influence the food system, and thereby the three aspects of food security (food utilization, food access and food availability) from a global political perspective. The three pillars of food security follow the World Food Summit (1996)'s definition (Thomas and Morrison, 2006) and the FAO added stability as the fourth pillar in 2001, which refers to the first three aspects over time. Since our model is static, we only focus on the first three pillars. We quantify food availability by food production, food access by food prices, wages and food demand, and food utilization by consumer surplus from food consumption. The political economy model follows the classic model of Grossman and Helpman (1994) and investigates the NPBT food policy effects on food security in a global context. We follow Weitzman (2001) and model the World Nation Official's (WNO, such as FAO) advice on global GM crop policies. The crucial assumption is that NPBT food regulations are supplementary to the regulations on non-NPBT food products and do not generate additional social benefits, such as higher levels of food or environmental safety. They are treated as safe as crops derived using “conventional” breeding. The WNO maximizes the sum of a weighted social welfare function and contributions from two lobby groups, an NPBT and a non-NPBT food group, who have contradictory interests toward the NPBT food technology. Some consumers have strong preferences for or against NPBT, while many other consumers are indifferent to either NPBT or non-NPBT food products or demand variety. Consequently, in the model we divide consumers into three groups (for, against, and indifferent). This helps us to integrate consumer preferences into the conflict of interest analysis.

We find that a stricter NPBT food regulation has negative effects on all three aspects of the global food security. This influence gets more negative when interests groups get involved. If the NPBT food technology is argued to be more efficient in production than the conventional technology, then the NPBT food lobby is more successful in the lobbying process when the WNO aims to improve the food security status. But if the non-NPBT food lobby group is very large, the NPBT food policy would stay strict. Therefore, the existence of a more powerful lobby group in the policy making process, be it the NPBT or the non-NPBT, makes that international organizations have difficulties in providing clear statements in favor of or against NPBTs, because these organizations depend on contributions from many sources.

## The model

We model the world as a closed economy, the world. There are two sectors in the economy, an agricultural food sector and a numeraire sector (*z*). Even though there are many farmers for NPBT and non-NPBT food production, we assume there are only two firms in the food sector in our model, a firm producing NPBT food *x*_*G*_ (henceforth: NPBT food firm, subscript *G*) and a firm producing non-NPBT food *x*_*N*_ (henceforth: non-NPBT food firm, subscript *N*). Labor and capital are the inputs for production^1^. The NPBT food firm uses the NPBT food technology as an additional input in its production process and receives benefits such as improved yield and/or reduced production costs, whereas the non-NPBT food firm only uses conventional agricultural technology for its production. The WNO, however, implements restrictions on the use of NPBT food technology to regulate NPBT ingredients, such as specific regulations for NPBT approval, regulations on cultivation, and private sector policies on NPBT-free food products. Coexistence policies, for instance, could require minimum distance, buffer zones, and/or rotation intervals when planting NPBT crops with reference to the conventional farming. Such regulations raise the cost of using NPBT food technology (Beckmann et al., 2006, 2011). We translate these policies into a single variable θ, (θ≥0), which represents an additional cost for the firm using the NPBT food technology; a stricter NPBT food policy means a higher NPBT food compliance cost.

We normalize the overall population to one and classify consumers into three types, denoted by superscripts α, β, γ, depending on their preferences. Fraction α of the population owns the NPBT food firm and shares the NPBT food profits. For example, NPBT food R&D researchers, producers, and retailers belong to this group. Consumers in this group have a strong preference for NPBT food and only consume NPBT food products. They are in favor of innovative technology and are convinced of its environmental and health benefits. Fraction β of the population belongs to the non-NPBT food group. It consists of people who own the non-NPBT food firm and earn the non-NPBT food profits. The anti-NPBT food organizations, conventional and organic food farmers, and anti-NPBT food consumers belong to this group. Consumers belonging to this group have a strong preference for non-NPBT food products and only purchase non-NPBT food products. The rest of consumers belong to fraction γ(= 1−α−β). This group considers NPBT and non-NPBT food products as imperfect substitutes. Its members do not worry much about the potential risks of the NPBT food technology; therefore, we label them henceforth as “indifferent”^2^. The two food firms, NPBT and non-NPBT, engage in Bertrand competition, that is, they compete for the γ consumers by setting a lower food price.

Consumers in the different groups purchase food products and numeraire goods subject to their income. Following Singh and Vives (1984), the quasi-linear utility functions of the three groups are^3^:

(1) $$\begin{array}{rc} U^{\alpha }=z^{\alpha }+ax_{G}^{\alpha }-\frac{1}{2}b{\left(x_{G}^{\alpha }\right)}^{2}\\ U^{\beta }=z^{\beta }+ax_{N}^{\beta }-\frac{1}{2}b{\left(x_{N}^{\beta }\right)}^{2}\\ U^{\gamma }\left(x_{G}^{\gamma },x_{N}^{\gamma }\right)=z^{\gamma }+ax_{G}^{\gamma }+ax_{N}^{\gamma }\\ - & \frac{1}{2}\left[b{\left(x_{G}^{\gamma }\right)}^{2}+2hx_{G}^{\gamma }x_{N}^{\gamma }+b{\left(x_{N}^{\gamma }\right)}^{2}\right]\\ s.tI^{i}=z^{i}+\underset{j}{\sum }p_{j}x_{j}^{i}fori=\alpha ,\beta ,\gamma andj=G,N \end{array}$$

where *z*^*i*^ is the utility from consuming the numeraire product with a price of one and *p*_*j*_ is the price of the food product. Given that *a, b* and *h* are positive parameters, we assume *b* > *h* > 0. For the indifferent food consumers, NPBT and non-NPBT are substitutes, when *h* = 1 they are perfect substitutes. γ consumers demand a mix of both NPBT food and non-NPBT food products. A price change of NPBT food has an effect on the demand for the non-NPBT food products by γ consumers. For α and β consumers, the total income consists of wage and a share of either NPBT or non-NPBT food profits. Consumers belonging to group γ only have income from their wages. The total demand for NPBT food products is $x_{G}^{\alpha }+x_{G}^{\gamma }$, and the total demand for non-NPBT food products is $x_{N}^{\beta }+x_{N}^{\gamma }$.

The NPBT food policy influence on the food market is modeled as a two-stage game. First, the WNO sets the NPBT policy level, and second the firms choose their prices. The NPBT and non-NPBT food firms have monopolies on their production. We use backward induction to identify the effects of the policy compliance cost. The firms' profits are:

(2) $$\begin{array}{r} \pi_{G}=p_{G}x_{G}-\left[w+\left(1+\theta \right)\phi r\right]x_{G} \end{array}$$

(3) $$\begin{array}{r} \pi_{N}=p_{N}x_{N}-\left(w+r\right)x_{N} \end{array}$$

where *p*_*i*_(*i* = *G, N*) is the price of either the NPBT or non-NPBT food product, *w* is the unit labor cost (wage rate), and *r* is the unit capital cost. ϕ is the productivity parameter of using NPBT technology, and 0 < ϕ < 1 represents the technology and is capital saving for food production. The unit costs for the NPBT and non-NPBT food firms are assumed to be independent of the level of output and are given by *w*+(1+θ)ϕ*r* and *w*+*r*.

In equilibrium, the NPBT food firm produces a sufficient quantity to meet the NPBT food demand, that is, $x_{G}=x_{G}^{\alpha }+x_{G}^{\gamma }$, and the non-NPBT food firm produces $x_{N}=x_{N}^{\beta }+x_{N}^{\gamma }$. The demand functions for both products are derived from consumers' maximization problems. These demand functions are (Appendix A):

$$\begin{array}{l} x_{G}=x_{G}^{\alpha }+x_{G}^{\gamma }=\frac{a-p_{G}}{b}+m-np_{G}+\delta p_{N}\\ =\frac{a}{b}+m-\left(\frac{1}{b}+n\right)p_{G}+\delta p_{N},\\ x_{N}=x_{N}^{\beta }+x_{N}^{\gamma }=\frac{a-p_{N}}{b}+c+\delta p_{G}-dp_{N}\\ =\frac{a}{b}+c-\left(\frac{1}{b}+n\right)p_{N}+\delta p_{G}. \end{array}$$

where *m* = (*ab*−*ah*)/(*b*^2^−*h*^2^), *n* = *b*^2^/(*b*^2^−*h*^2^), δ = *h*/(*b*^2^−*h*^2^). Using the demand functions, we can solve for the reaction functions of the firms (see Appendix B):

$$\begin{array}{l} p_{G}=\frac{\frac{a}{b}+m+\delta p_{N}+\left(\frac{1}{b}+n\right)\left[w+\left(1+\theta \right)\phi r\right]}{2\left(\frac{1}{b}+n\right)}\text{and}\\ p_{N}=\frac{\frac{a}{b}+m+\delta p_{G}+\left(\frac{1}{b}+n\right)\left(w+r\right)}{2\left(\frac{1}{b}+n\right)}. \end{array}$$

Using these we can solve for the equilibrium price for the NPBT food product:

$$\begin{array}{l} p_{G}^{*}=\frac{1}{b^{2}\delta^{2}-4b^{2}n^{2}-8nb-4}\\ \left(\begin{array}{c} -2{\left(1+bn\right)}^{2}\left(w+\left(1+\theta \right)\phi r\right)\\ -b\delta \left(\left(1+bn\right)\left(w+r\right)+\left(bm+a\right)\right)\\ -2\left(a+bm\right)\left(1+bn\right) \end{array}\right), \end{array}$$

where $\partial p_{G}^{*}/\partial \theta >0$. The NPBT food compliance cost influences the NPBT food price directly, and the non-NPBT food price indirectly. We solve for the equilibrium non-NPBT food price from the reaction function and find $\partial p_{N}^{*}/\partial \theta >0$, but $\partial p_{G}^{*}/\partial \theta >\partial p_{N}^{*}/\partial \theta$. The NPBT food firm prefers a low NPBT food policy cost and more NPBT food technology, whereas the non-NPBT food firm prefers a high NPBT food price to attract more γ consumers to purchase non-NPBT food products.

The inverse demand functions for food products are: $p_{G}^{\alpha }=a-bx_{G}^{\alpha }$ for the NPBT food consumers, $p_{N}^{\beta }=a-bx_{N}^{\beta }$ for the non-NPBT food consumers, and $p_{G}^{\gamma }=a-bx_{G}^{\gamma }-hx_{N}^{\gamma }$ and $p_{N}^{\gamma }=a-hx_{G}^{\gamma }-bx_{N}^{\gamma }$ for γ consumers. In the equilibrium, the consumer surplus is $cs_{G}^{\alpha }=\overset{x_{G}^{\alpha *}}{\underset{0}{\int }}p\left(x_{G}^{\alpha }\right)dx_{G}^{\alpha }-p_{G}^{*}x_{G}^{\alpha *}$ for α consumers and $cs_{N}^{\beta }=\overset{x_{N}^{\beta *}}{\underset{0}{\int }}p\left(x_{N}^{\beta }\right)dx_{N}^{\beta }-p_{N}^{*}x_{N}^{\beta *}$ for β consumers. γ consumers demand both NPBT and non-NPBT food products, so $cs^{\gamma }=cs_{G}^{\gamma }+cs_{N}^{\gamma }=\overset{x_{G}^{\gamma *}}{\underset{0}{\int }}p\left(x_{G}^{\gamma }\right)dx_{G}^{\gamma }-p_{G}^{*}x_{G}^{\gamma *}+\overset{x_{N}^{\gamma *}}{\underset{0}{\int }}p\left(x_{N}^{\gamma }\right)dx_{N}^{\gamma }-p_{N}^{*}x_{N}^{\gamma *}$. The aggregate social welfare of each group is given by:

(4) $$\begin{array}{r} W^{\alpha }=\pi_{G}\left(\theta \right)+cs_{G}^{\alpha }\left(\theta \right)\\ W^{\beta }=\pi_{N}\left(\theta \right)+cs_{N}^{\gamma }\left(\theta \right)\\ W^{\gamma }=cs_{G}^{\gamma }\left(\theta \right)+cs_{N}^{\gamma } \end{array}$$

Aggregate social welfare is the sum of the three groups' welfare in Equation (4):

(5) $$\begin{array}{r} W\left(\theta \right)=\pi_{G}\left(\theta \right)+\pi_{N}\left(\theta \right)+cs_{G}\left(\theta \right)+cs_{N}\left(\theta \right) \end{array}$$

Thus, we can find the socially optimal NPBT food regulation by letting

(6) $$\begin{array}{r} \frac{\partial W\left(\theta \right)}{\partial \theta }=\frac{\partial W^{\alpha }\left(\theta \right)}{\partial \theta }+\frac{\partial W^{\beta }\left(\theta \right)}{\partial \theta }+\frac{\partial W^{\gamma }\left(\theta \right)}{\partial \theta }=0 \end{array}$$

## NPBT food policy effects on food security

We investigate the NPBT food regulation effects on availability, access and utilization of food security. Food security is a multi-aspects issue. To obtain specific results, we interprete the three dimensions of food security in our model in economic terms. As we stated earlier, the change of NPBT food regulation influences the NPBT food group directly and the non-NPBT food group indirectly. In addition, it influences the consumption distribution across NPBT and non-NPBT food products for the indifferent consumers. The marginal effects in Table 1 (derivation: Appendix C) shows the NPBT food policy effects.

**Table 1:** Marginal policy effects due to an increase in regulation on food security.

	**Availability**	**Access**			**Utilization**	
	**Production**	**Price**	Total income^*^	**Demand**	**Total Utility**	**Consumer surplus**
NPBT	−	+	−	−	−	−
Non-NPBT	+	+	+	+	+	−
Indifferent	N/A	N/A	N/A	−	−	−
Total	−	+	−/+	−	+/−	−

“−” denotes a decrease and “+” an increase (see Appendix C);

* *Total income constitutes profits and wages*.

In Table 1, food availability is the production of food in the economy, i.e., *x*_*G*_+*x*_*N*_. A stricter NPBT food policy will reduce the production of NPBT food products because a higher NPBT food regulation compliance cost increases the price of capital input for the NPBT firm. As a result the price of NPBT food products increases and consequently the NPBT food demand from the α and γ group decreases. The non-NPBT food demand from the β group is not influenced by the NPBT food policy change, but if the demand for NPBT food products from the indifferent group decreases, the demand for non-NPBT food products will increase. Hence, a change of the NPBT food policy level has an indirect effect on the non-NPBT food demand. There are two opposing policy effects on both the NPBT and non-NPBT food production, but the policy effect on the overall food production is negative. The reason is that a higher NPBT food policy cost directly decreases the demand of both the NPBT food consumers and a portion of the indifferent consumers, which outweighs the positive effect on the non-NPBT food production, which is driven by only a part of the indifferent consumers.

The NPBT food regulation influences food access, which includes food affordability, food allocation, and consumer choices. We quantify the food access by food prices, the total income of consumers, and their food demand. The NPBT food consumers are directly influenced by the change of NPBT food price and income. If the NPBT food compliance cost increases, the NPBT food price increases, hence more indifferent consumers choose non-NPBT food. The increasing demand for non-NPBT food drives the non-NPBT food price up. The NPBT food firm's profit decreases under a stricter NPBT food policy defined in Equation (2), but the non-NPBT food profit increases from a higher demand and the resulting higher equilibrium price of non-NPBT food products. Wage rate does not change, so the total income is smaller for the NPBT food group, larger for the non-NPBT food group and the same for the indifferent group. The price increase of both NPBT and non-NPBT decreases the average households' affordability and access to food.

Food utilization comprises nutritional value, social value, and food safety. We measure this by total food demand and consumer surplus from food consumption. Consumers choose food products according to their preferences (see Equation 1); furthermore, they believe the food they choose is of higher value. Higher NPBT food regulation costs decrease the NPBT food production and total income of the NPBT food group and the demand for NPBT food products, hence nutrient intake decreases. If the NPBT food policy becomes stricter, consumer surplus of the NPBT food group will be reduced as well. The non-NPBT food consumers also lose from a higher non-NPBT food price induced by a higher demand from the indifferent group. Since the policy effect on the NPBT food price is larger than on the non-NPBT food price and the effect on the NPBT food production is opposing that on the non-NPBT food production, the policy effect on the total consumer surplus is negative.

Thus, we conclude that

**Proposition 1** *A more stringent NPBT food regulation has a negative impact on global food security measured by its influence on food availability, accessibility and utilization*.

## The political process

**Figure 2:**
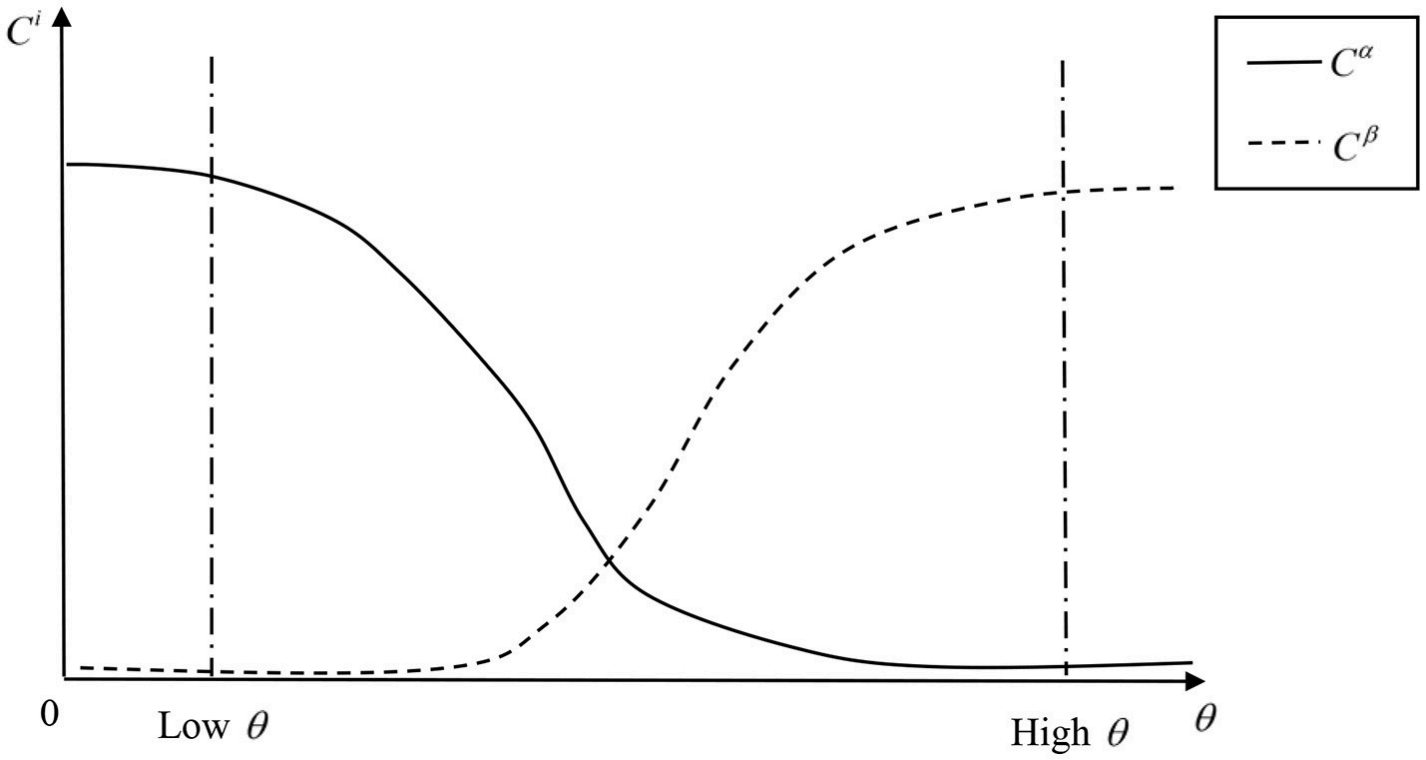
Truthful Contribution Schedules and the Level of GM Food Regulations.

We endogenize the NPBT food policy in the policy-making process. The NPBT and non-NPBT food groups have opposing interests toward the level of NPBT food policy. The NPBT food group lobbies for lower NPBT food regulation costs in order to reduce the NPBT food firm's production costs, whereas the non-NPBT food group lobbies for a stricter NPBT food regulation. Members in either NPBT or non-NPBT group have strong incentive to lobby, whereas those who have incentive to “free-ride” on the efforts of others are consumers in the indifferent group in the model (Olson, 1971). The indifferent group does not make any contribution to the WNO. Lobby groups influence the regulation in several ways. For example, they can make contributions, endorsements and committed votes to the WNO so as to influence the policy outcome. For simplicity, we model these contributions as monetary equivalents from the interest groups. We follow Grossman and Helpman (1994)'s model and define the WNO payoff function as a maximization of a weighted sum of aggregate social welfare plus contributions from the lobbies. The WNO payoff is given by:

(7) $$\begin{array}{r} G\left(\theta ;C^{\alpha },C^{\beta }\right)=qW\left(\theta \right)+\left(1-q\right)\left[C^{\alpha }\left(\theta \right)+C^{\beta }\left(\theta \right)\right] \end{array}$$

where *q* is the weight parameter, 0 < *q* < 1, that the WNO attaches to the social welfare. *C*^α^(θ) and *C*^β^(θ) are the differentiable truthful contribution schedules of the two lobbying groups like in Grossman and Helpman (1994), which means the NPBT food policy effects on the groups' contribution always represent the lobbies' policy preferences. We show this with two levels (high and low) of NPBT food regulations in Figure 2. For example, the negative effects resulting from higher NPBT food regulation costs induce the NPBT food lobby to contribute less. The non-NPBT food contribution reaches the maximum at a high level of NPBT food regulation. The maximum contribution that any lobby can make is its gross income, which include wages and firms' profits.

The political process is a three-stage non-cooperative game. Two lobbies simultaneously announce their contribution schedules to the WNO in the first stage, and the WNO decides the NPBT food policy that maximizes its payoff in the second stage. In the third stage, firms choose prices and lobbies pay their contributions.

The NPBT and non-NPBT food groups make the total contribution *B*^*i*^(θ) from their income for lobbying. The amount of the contribution from each group depends on the number of consumers and the share of their donations. The net income of each group is their gross income minus the lobbying costs:

(8) $$\begin{array}{r} I_{P}^{\alpha }=\alpha wL+\pi_{G}\left(\theta \right)-B^{\alpha }\left(\theta \right)\\ I_{P}^{\beta }=\beta wL+\pi_{N}\left(\theta \right)-B^{\beta }\left(\theta \right)\\ I^{\gamma }=\gamma wL \end{array}$$

where $I_{P}^{i}\left(i=\alpha ,\beta \right)$ denotes the group's net income in the political game. The indifferent group does not lobby, they only choose the food product available in the market, so their net income does not change. We assume that lobbying is costly (Laffont and Tirole, 1993); and that a one dollar contribution costs (1+λ^*i*^) dollars in donations for lobby *i*. That is, *B*^*i*^(θ) = (1+λ^*i*^)*C*^*i*^(θ), where λ^*i*^ is nonnegative and represents the efficiency of lobbying. *B*^*i*^ is the total money collected for lobbying from group members. A group with a large membership collects a higher sum of contributions. But the lobbying efficiency also matters for the political outcomes. A higher λ^*i*^ implies less efficient lobbying or, equivalently, higher lobbying cost. The WNO may have different preferences for interest groups. One group may have a higher efficiency and hence lower costs than the other group in the lobbying process. Lemma 2 of Grossman and Helpman (1994) provides the micro-foundations for lobbying and implies the optimal contribution level *C*^i*^(θ) for each group, which is determined by:

(9) $$\begin{array}{r} \frac{\partial W^{i}\left(\theta \right)}{\partial \theta }=\left(1+\lambda^{i}\right){{\frac{\partial {C^{i}}^{*}\left(\theta \right)}{\partial \theta }}^{\;}}^{\;}fori=\alpha ,\beta \end{array}$$

In the above equation, we can see that due to lobbying costs (λ^*i*^), the marginal effect of NPBT food policy on the contribution is smaller than the marginal effect of NPBT food policy on welfare. It is, therefore, costly to lobby. The optimal political NPBT food policy is determined by:

(10) $$\begin{array}{r} \frac{\partial G\left(\theta \right)}{\partial \theta }=q\frac{\partial W\left(\theta \right)}{\partial \theta }+\left(1-q\right)\left[\frac{\partial C^{\alpha }\left(\theta \right)}{\partial \theta }+\frac{\partial C^{\beta }\left(\theta \right)}{\partial \theta }\right]=0 \end{array}$$

We substitute Equation (9) into Equation (10), and find that the first order condition for NPBT food policy can be expressed as:

(11) $$\begin{array}{r} \frac{\partial G\left(\theta \right)}{\partial \theta }=\left(\frac{1-q}{1+\lambda^{\alpha }}+q\right)\frac{\partial W^{\alpha }\left(\theta \right)}{\partial \theta }+\left(\frac{1-q}{1+\lambda^{\beta }}+q\right)\\ \frac{\partial W^{\beta }\left(\theta \right)}{\partial \theta }+q\frac{\partial W^{\gamma }\left(\theta \right)}{\partial \theta }=0 \end{array}$$

Equation (11) is different from Equation (6), which means that the politically determined NPBT food policy is a deviation from the social optimum, unless λ^*i*^ is extremely high or *q* = 1. We can see that lobby groups will not make contributions if the lobbying is extremely costly (i.e., λ^*i*^ is high). Similarly, the WNO will not consider the contribution from groups if it only considers welfare (i.e., *q* = 1).

Lobbying influences NPBT food policy and the food security status because the lobby contribution is taken from the income, according to Equation (8). The two groups spend *B*^*i*^ for lobbying, so the budget constraint shifts inward, which decreases the demand for both NPBT and non-NPBT food products as well as numeraire goods. The inwardly shifting budget constraint directly influences food security due to food access. The reduction in food demand decreases the amount of food consumed in equilibrium. More lobbying efforts from the NPBT food lobby may push the NPBT food compliance cost down and improve the overall food security, but food security will be improved only if the benefits from lower policy costs compensate the lobbying costs of the two groups. But, if the policy is stricter under lobbying, the food security will decline. To summarize,

**Lemma 1** *The politically determined NPBT food regulation is a deviation from the socially optimal NPBT food regulation due to the unbalanced lobbying power of interest groups. Political rivalry among interest groups worsens the food security status unless the benefit from a lenient NPBT food regulation compensates for the lobbying costs*.

## Food security as a policy target

NPBT food technology is a possible solution to improve food productivity and security (e.g., Paarlberg, 2010; Vigani and Olper, 2013). In this section, we discuss the political rivalry concerning NPBT food policy formation if the WNO wants to improve the food security level. We aggregate the three food security aspects (availability, access, and utilization) into a single variable *s*, which denotes the world's food security level.

Suppose the world has a target food security level to reach and allows NPBT food technology to be used in the agricultural food production. If the food security level is below the target level, the WNO would like to increase its food output by using more of the productive technology. Therefore, we define $\mu =\bar{s}/s$, where $\bar{s}$ is the target food security level and *s* is the current level. $0<\bar{s}<1$ and 0 < *s* < 1. We use μ to indicate the inverse of the current progress toward the food security level of the world, $\bar{s}$. The non-NPBT food consumers constitute a significant part of social welfare, but an increase of *s* through more NPBT food input does not increase their welfare directly. Therefore, we use an indirect way of including food security in the WNO's objective function, namely by changing the weights of the different lobbying contributions based on progress toward food security. Although μ is an exogenous variable for the lobbying groups and does not depend on the groups' lobbying efforts, it will influence the lobbies' contribution behaviors in the political process. In this case, the WNO payoff function becomes

(12) $$\begin{array}{r} G_{s}=qW+\left(1-q\right)\left(\mu C^{\alpha }+C^{\beta }\right) \end{array}$$

We can use backward induction to find the optimal lobbying schedule for the two lobby groups. If the NPBT food lobbying group knows that the WNO will try to increase the food security level in the second stage, it will change its optimal lobbying schedule in the first stage. That is,

(13) $$\begin{array}{r} \frac{\partial W^{\alpha }}{\partial \theta }=\left(\frac{1+\lambda_{\;}^{\alpha }}{\mu }\right)\frac{\partial C_{s}^{\alpha }}{\partial \theta } \end{array}$$

The NPBT food group spend $B_{s}^{\alpha }=\left(\left(1+\lambda_{\;}^{\alpha }\right)/\mu \right)C_{s}^{\alpha }$ for lobbying, which is smaller than in the absence of a food security improvement target (section The Political Process). The NPBT food group contribution weighs more in the policy-making process when μ>1 (i.e., food insecure). NPBT food consumers spend less of their income, which improves food affordability under a constant NPBT food price of food consumption.

Comparing Equation (13) with (9), we can see that the NPBT food group is more efficient in the political process. One unit of welfare gain in the lobbying process needs $\left(1+\lambda_{\;}^{\alpha }\right)/\mu$ units of contribution instead of (1+λ^α^). From the WNO perspective, the income from lobby groups stays constant because one unit of NPBT food group contribution counts for more in the WNO payoff function. Lobby groups would spend less than when food security is not a policy issue for a lenient NPBT food regulation, according to Equation (13).

From the above discussion, we determine that

**Lemma 2** *When the food security status is an important part of a WNO policy, the NPBT food group will be more efficient in the political game, but the WNO will not be worse off because it has the same total contribution income*.

When the production level reaches its target food security level, the WNO resorts to its old weights. In this case, the WNO does not weigh the NPBT food lobby heavier than the non-NPBT food lobby; lobbies compete equally in the policy game. If the non-NPBT food lobby has a large membership and is more powerful in the political process than the NPBT food lobby, the non-NPBT food contribution will be high, and finally the NPBT food regulation will be strict. In this case, the WNO could also weight the non-NPBT food lobby heavier than the NPBT food lobby without decreasing its payoff.

## Discussion of implications

The results of the model show more strict regulations on the approval and use of NPBTs will have negative implications for food security following standard definitions of food security. The costs of food production increase by more stringent regulations decreasing the overall supply of food. Further, the fact that decision makers are exposed to lobbying and lobby groups can influence NPBT regulation. This may seem rather trivial, but the important message is that lobbying is not only done by one group. The more policy makers consider implications for food security, the less they will be influenced by lobby groups. In the case of NPBTs, the implication is that supporters of the technology have to lobby less than opponents or if they lobby they will stress the importance of NPBTs for food security.

One of the important assumption being made is that NPBTs provide an improvement in crop yield and increase food security. Readers have to bear in mind that this is one of the important assumptions in our model and further discussions rely on that assumption. The applications of NPBTs, however, suggest this will indeed be the case. Some of the already available applications include herbicide resistant oilseed rape and sunflower cultivated in France, non-browning apples, mushrooms and potatoes, late-blight resistant potatoes and more (Sprink et al., 2016; CAST, 2018). It is reasonable to expect the use of NPBTs will generate environmental as well economic benefits similar to GMOs increasing food security via higher yields and safer food. As the discussion about the safety of NPBTs shows, this is for most cases a reasonable assumption (Sprink et al., 2016).

Many low food security countries often implement strict food policies for GMOs (e.g., Paarlberg, 2009; Wesseler et al., 2017). Similar results can be expected for NPBTs. The results of our model suggest that policy makers are strongly influenced by lobby groups and that in the context of GMOs anti GMO lobby groups have been more successful. This supports the argument made by Paarlberg (2009) that some policy makers in Africa orient their policies more toward the policies in the European Union than being guided by the needs of their own populations. Similar observations have been reported for the case of insect resistant cotton (Herring, 2008) and Vitamin A enriched rice (Wesseler and Zilberman, 2014) in India.

For the case of NPBTs the possibility exists that the outcome for the case of GMOs can be changed if supporters for NPBTs increase their lobbying efforts and combine this with stressing the importance for food security. We use the parable of a World Nation Official as a benevolent dictator that has the power to decide about regulatory policies. The more food security will be considered as being important by the WNO the less influential lobby groups trying to block the introduction of NPBTs will be. Food security has become an important policy agenda item as part of the Sustainable Development Goals (SDGs) under Goal 2: end hunger, achieve food security and improved nutrition and promote sustainable agriculture. This increases the possibility that food security will receive more attention than before and according to our model results, the impact of lobby groups blocking the use of NPBTs will be reduced.

Looking at the European Union where there is an on-going debate about the regulation of NPBTs the results of our model provide some important insights. First, groups gaining and losing from NPBTs will both lobby and try to influence the policy outcome. Many environmental groups oppose the use of NPBTs and lobby for regulations similar to regulations for GMOs (Smart et al., 2015; Sprink et al., 2016; Purnhagen et al., 2018a). Their impact on regulatory policies in the EU can be expected to be stronger as in comparison to their impact on policies at e.g., FAO as decision making bodies within the European Union can be expected to care less about food security considering the supply of food within the European Union, relatively speaking. This finds support by the recent judgment of the Court of Justice of the European Union (CJEU, 2018).

The challenge for regulators is to take the implications of their regulatory policies for food security into considerations. A more stringent regulatory system reduces food security under the assumption of food safety. A stringent regulatory policy not only includes the requirements for safety assessments, which can already be substantial (e.g., Smyth et al., 2017), but also the time-length (Smart et al., 2017). There exist a number of opportunities in the European Union and the United States for improving regulatory policies (Wesseler and Kalaitzandonakes, 2011; CAST, 2018; Purnhagen et al., 2018b). A clear regulatory policy that aims at reducing regulatory costs without compromising food safety can have a positive “lobbying” effect for policy makers in particular in Africa who look for guidance and are exposed to different lobby groups (Falck-Zepeda et al., 2013).

## Conclusion

NPBTs are an advanced technology to improve agricultural production. They are regarded as one of the options for improving global food security. The dispute about the effects of the technology on humans and nature impede its application as e.g., for the case of Vitamin A enriched rice (Wesseler and Zilberman, 2014). This debate also applies to NPBTs and as a consequence the level of NPBT food regulation is also a political game.

This paper develops a standard political economy model of NPBT regulations, modeling the NPBT food policy as the outcome of a NPBT and non-NPBT food group lobbying game. We find that a stricter NPBT food policy has negative effects on three aspects of food security: availability, access, and utilization. The politically determined NPBT food policy worsens the food security situation under the costly lobbying assumption. We also discuss when the WNO weighs the NPBT and non-NPBT food lobbies' contributions differently depending on the food security status. The NPBT food lobby becomes more efficient in the political game than the non-NPBT food group when the WNO commits to improving food security. If the non-NPBT food lobby is large and strong, it will make large lobbying contributions for a stricter NPBT food policy, even when the world is food insecure. The pro-NPBT food lobby group will be more effective if the WNO policy reflects concerns about food security. Linking the results to international debates on NPBTs in the case where the opposition to the NPBT food technology is more successful, either the opposition has more financial resources available for lobbying or the governing bodies are less concerned about food security. What in this case is the most dominating factor will be an empirical question. Considering the importance of the issue, this warrants further research.

High-income countries, such as some European countries, can afford to implement a strict NPBT food policy without worsening the food security condition, but for more than two-thirds of low-middle-income countries, the food security issue remains (Economist Intelligence Unit, 2015). The NPBT food policy in many developing countries, such as Southern Asian and African countries, is still under debate, whereas many of them experience food shortages and malnutrition. The various countries could have tailored NPBT food policies according to each of their domestic food supply and demand, but they also need to take food security into consideration while making food policies.

The model presented provides an economic explanation for the observed lobbying activities. For many, it is obvious that the input supply sector of the technology will gain from lobbying for less strict regulations. But, there are also some private gains from lobbying against the technology, as claimed explicitly by Apel (2010) and more indirectly by Paarlberg (2009), when referring to projects funding biotechnology regulations in Africa. The political rivalry between contradictory interest groups offers an additional explanation why new technologies often have faced resistance, not only GMOs (Juma, 2016; Moses, 2016). Our model explains the market competition of the NPBT and non-NPBT food products and the driving force of lobbying competition that drives opposition to new technologies. The challenge is to identify what are the private economic gains of lobby groups that oppose new technologies. One obvious benefit is reducing stakeholder losses from being displaced by the new technologies. In the case of NPBT food technologies, environmental and other non-governmental organizations are more vocal within the European Union. Within the European Union, the group for non-NPBT food products is much larger than the group supporting the NPBT food technology (Clancy, 2017). Again, this raises the question of what do they gain? Or do EU policy makers care less about food security?

## Author contributions

QS, MP, and JW contributed conception and design of the study. QS developed the model and simulation. QS and MP developed the game theoretical model. QS wrote the first draft of the manuscript. QS, MP, and JW wrote sections of the manuscript. All authors contributed to manuscript revision, read and approved the submitted version.

### Conflict of interest statement

The authors declare that the research was conducted in the absence of any commercial or financial relationships that could be construed as a potential conflict of interest.

## Footnotes

^1^We only focus on the agricultural sector and take the labor and capital price exogenous.

^2^γ consumers are indifferent to the NPBT food technology, not the NPBT or the non-NPBT food products.

^3^We assume the same parameters *a* and *b* for the three groups, because i) all consumers demand food products, no matter NPBT food or non-NPBT, ii) we want to simplify the analytical calculation and identify the policy effects.
