# Supplementary material for: New Plant Breeding Techniques Under Food Security Pressure and Lobbying
Source: Front Plant Sci. 2018 Sep 19;9:1324. doi: 10.3389/fpls.2018.01324 (PMC6156281; doi:10.3389/fpls.2018.01324)
Supplement: Supplementary file 1 [file Presentation_1.pdf]

Appendix

A. Derivation of demand functions of the three types of consumers:

NPBT food consumers maximize: $U^{\alpha} = z^{\alpha} + ax_G^{\alpha} - \frac{1}{2}b(x_G^{\alpha})^2$

Non-NPBT food consumers maximize: $U^{\beta} = z^{\beta} + ax_N^{\beta} - \frac{1}{2}b(x_N^{\beta})^2$

Indifferent consumers maximize:

$$U^{\gamma}\left(x_G^{\gamma}, x_N^{\gamma}\right) = z^{\gamma} + ax_G^{\gamma} + ax_N^{\gamma} - \frac{1}{2}\left[b\left(x_G^{\gamma}\right)^2 + 2hx_G^{\gamma}x_N^{\gamma} + b\left(x_N^{\gamma}\right)^2\right]$$

Subject to their respective incomes:

$$I^{\alpha} = w\alpha L + \pi_G$$
$$I^{\beta} = w\beta L + \pi_N$$
$$I^{\gamma} = w\gamma L$$

where b is positive, $b^2 - h^2 > 0$ and $ab - ah > 0$ given that $h > 0$, NPBT and non-NPBT food are imperfect substitutes. A price change of NPBT food products has effects on the total demand for the non-NPBT food products.

Setting up the maximization problems subject to the budget constraints we can solve for the demand functions of the different groups:

$$x_G^{\alpha} = \frac{a - p_G}{b}$$
$$x_N^{\beta} = \frac{a - p_N}{b}$$

For the indifferent consumers:

From the first-order condition for the utility function of the γ consumers, we find:

$$p_G = a - bx_G^{\gamma} - hx_N^{\gamma}$$
$$p_N = a - hx_G^{\gamma} - bx_N^{\gamma}$$

and solving these for the direct demand gives:

$$x_G^\gamma = m - np_G + \delta p_N$$
$$x_N^\gamma = m + \delta p_G - np_N$$

where

$m = \dfrac{ab - ah}{b^2 - h^2}, n = \dfrac{b}{b^2 - h^2}, \delta = \dfrac{h}{b^2 - h^2}$, given our earlier assumptions about parameters, these imply that: $n > 0$, $\delta > 0$, $n > \delta$, $n^2 > \delta^2$.

From the direct demand functions, we can see there are cross-price effects.

Total demand for both products are then respectively:

$$x_G = x_G^\alpha + x_G^\gamma = \frac{a - p_G}{b} + m - np_G + \delta p_N = \frac{a}{b} + m - \left(\frac{1}{b} + n\right) p_G + \delta p_N$$

$$x_N = x_N^\beta + x_N^\gamma = \frac{a - p_N}{b} + m + \delta p_G - np_N = \frac{a}{b} + m - \left(\frac{1}{b} + n\right) p_N + \delta p_G .$$

B. Solving for the equilibrium prices:

Profit function of the NPBT food firm:

$$\pi_G = p_G x_G - \left[w + (1+\theta)\phi r\right] x_G$$

Inserting the total demand function for NPBT food products and taking the first derivative, and solving for p_G gives the reaction function of the NPBT food firm:

$$\frac{\partial \pi_G}{\partial p_G} = \frac{a}{b} + m - 2\left(\frac{1}{b} + n\right) p_G + \delta p_N + \left(\frac{1}{b} + n\right)\left[w + (1+\theta)\phi r\right] = 0$$

$$p_G = \frac{\frac{a}{b} + m + \delta p_N + \left(\frac{1}{b} + n\right)\left[w + (1+\theta)\phi r\right]}{2\left(\frac{1}{b} + n\right)}$$

Similarly, the profit function of the non-GM food firm:

$$\pi_N = p_N x_N - (w + r) x_N$$

Inserting the total demand function for non-NPBT food products and taking the first derivative, and solving for p_N gives the reaction function of the non-NPBT food firm:

$$\frac{\partial \pi_N}{\partial p_N} = \frac{a}{b} + m - 2\left(\frac{1}{b} + n\right) p_N + \delta p_G + \left(\frac{1}{b} + n\right)(w + r) = 0$$

$$p_N = \frac{\frac{a}{b} + m + \delta p_G + \left(\frac{1}{b} + n\right)(w + r)}{2\left(\frac{1}{b} + n\right)}$$

By inserting the reaction function of the non-NPBT food firm in the function of the NPBT food firm, we can solve for the equilibrium price p_G^*

$$p_G^* = \frac{1}{b^2\delta^2 - 4b^2n^2 - 8nb - 4}\begin{pmatrix} -2(1+bn)^2\left(w + (1+\theta)\phi r\right) \\ -b\delta\left((1+bn)(w+r) + (bm + a)\right) \\ -2(a + bm)(1+bn) \end{pmatrix}$$

So, $\frac{\partial p_G^*}{\partial \theta} = \frac{\overbrace{-2(1+bn)^2 \phi r}^{<0}}{\underbrace{b^2\left(\delta^2 - 4n^2\right) - 8nb - 4}_{<0}} > 0$, for $n > \delta$.

Using the reaction function of the non-NPBT food firm we find for the equilibrium non-NPBT food price:

$\frac{\partial p_N^*}{\partial \theta} = \frac{\delta}{2\left(\frac{1}{b} + n\right)} \frac{\partial p_G}{\partial \theta} > 0$. We find $\frac{\delta}{2\left(\frac{1}{b} + n\right)} < 1$, for $n > \delta$, so $\frac{\partial p_G^*}{\partial \theta} > \frac{\partial p_N^*}{\partial \theta}$.

C. The marginal effects of the NPBT food policy on food security:

Food availability: quantity (production):

Given that in equilibrium the quantity on the market is solely determined by equilibrium prices we can use the demand functions to establish the effects on production:

$$\frac{\partial x_G^*}{\partial\theta} = -\left(\frac{1}{b}+n\right)\frac{\partial p_G}{\partial\theta} + \delta\frac{\partial p_N}{\partial\theta} = -\left(\frac{1}{b}+n\right)\frac{\partial p_G}{\partial\theta} + \delta\frac{\partial p_N}{\partial p_G}\frac{\partial p_G}{\partial\theta}$$

$$= -\left(\left(\frac{1}{b}+n\right) - \frac{\delta^2}{2\left(\frac{1}{b}+n\right)}\right)\frac{\partial p_G}{\partial\theta} = -\underbrace{\left(\frac{\overbrace{2\left(\frac{1}{b}+n\right)^2 - \delta^2}^{>0,\text{ for } n>\delta}}{2\left(\frac{1}{b}+n\right)}\right)}_{<0}\frac{\partial p_G}{\partial\theta} < 0$$

There is no direct influence on the price and quantity of non-NPBT food products from the NPBT food policy, but given that the two are imperfect substitutes we have

$x_N\left(p_G(\theta), p_N\left(p_G(\theta)\right)\right)$, and $\frac{\partial x_N}{\partial p_G} > 0$,

$$\frac{\partial x_N^*}{\partial\theta} = -\left(\frac{1}{b}+n\right)\frac{\partial p_N}{\partial\theta} + \delta\frac{\partial p_G}{\partial\theta} = -\left(\frac{1}{b}+n\right)\frac{\partial p_N}{\partial p_G}\frac{\partial p_G}{\partial\theta} + \delta\frac{\partial p_G}{\partial\theta}$$

$$= -\left(\left(\frac{1}{b}+n\right)\frac{\delta}{2\left(\frac{1}{b}+n\right)} - \delta\right)\frac{\partial p_G}{\partial\theta} = -\underbrace{\left(\frac{\delta}{2}-\delta\right)}_{>0}\frac{\partial p_G}{\partial\theta} > 0 \quad .$$

For the policy effect on the total food supply:

$$\frac{\partial x_G^*}{\partial\theta} + \frac{\partial x_N^*}{\partial\theta} = -\left(\frac{2\left(\frac{1}{b}+n\right)^2 - \delta^2}{2\left(\frac{1}{b}+n\right)}\right)\frac{\partial p_G}{\partial\theta} - \left(\frac{\delta}{2}-\delta\right)\frac{\partial p_G}{\partial\theta} = \left(\frac{-2\left(\frac{1}{b}+n\right)^2 + \delta^2 + \delta\left(\frac{1}{b}+n\right)}{2\left(\frac{1}{b}+n\right)}\right)\frac{\partial p_G}{\partial\theta}$$

$$= \left(\frac{\overbrace{\frac{1}{b}(\delta-4n)}^{<0, n>\delta} \overbrace{-2\left(\frac{1}{b}\right)^2}^{<0} \overbrace{(\delta-n)(\delta+2n)}^{<0,\ n>\delta}}{2\left(\frac{1}{b}+n\right)}\right)\frac{\partial p_G}{\partial\theta} < 0$$

Food access: food prices, income, and demand:

NPBT food price: $\frac{\partial p_G^*}{\partial\theta} > 0$

Non-NPBT food price has no direct effects, but from the reaction function,

$$\frac{\partial p_N^*}{\partial \theta} = \frac{\partial p_N^*}{\partial p_G^*}\frac{\partial p_G^*}{\partial \theta} > 0 .$$

Wage income is determined by the whole economy, the labor supply, and the demand from the food market and the numeraire market, so we assume it is exogenous and constant. However, the NPBT and non-NPBT food profits are influenced.

$$\frac{\partial \pi_G^*}{\partial \theta} = -r x_G^* < 0 ,$$

$$\frac{\partial \pi_N^*}{\partial \theta} = \frac{\partial p_N^*}{\partial \theta} x_N^* + \underbrace{\left(p_N - w - r\right)}_{>0}\frac{\partial x_N^*}{\partial \theta} > 0 .$$

The profit effects depend on both the NPBT and non-NPBT food productions, so the sign it hard to say.

Marginal effects on demands:

$$\frac{\partial x_G^\alpha}{\partial \theta} = -\frac{1}{b}\frac{\partial p_G}{\partial \theta} < 0$$

$$\frac{\partial x_N^\beta}{\partial \theta} = -\frac{1}{b}\frac{\partial p_N}{\partial \theta} < 0 ,$$

$$\frac{\partial x_G^\gamma}{\partial \theta} = -n\frac{\partial p_G}{\partial \theta} + \delta\frac{\partial p_N}{\partial \theta} = -n\frac{\partial p_G}{\partial \theta} + \delta\frac{\partial p_N}{\partial p_G}\frac{\partial p_G}{\partial \theta} = -n\frac{\partial p_G}{\partial \theta} + \frac{b\delta^2}{2\left(1+bn\right)}\frac{\partial p_G}{\partial \theta}$$
$$= \left(-n + \frac{\delta^2}{2\left(\frac{1}{b}+n\right)}\right)\frac{\partial p_G}{\partial \theta} = \underbrace{\left(\frac{-2n\frac{1}{b} - 2n^2 + \delta^2}{2\left(\frac{1}{b}+n\right)}\right)}_{<0}\frac{\partial p_G}{\partial \theta} < 0 \qquad ,$$

for $b^2 - h^2 > 0$

$$\frac{\partial x_N^\gamma}{\partial \theta} = \delta\frac{\partial p_G}{\partial \theta} - n\frac{\partial p_N}{\partial p_G}\frac{\partial p_G}{\partial \theta} = \left(\delta - n\frac{\delta}{2\left(\frac{1}{b}+n\right)}\right)\frac{\partial p_G}{\partial \theta} = \left(\frac{2\delta\frac{1}{b} + \delta n}{2\left(\frac{1}{b}+n\right)}\right)\frac{\partial p_G}{\partial \theta} > 0 .$$

$$\frac{\partial x_G^\gamma}{\partial\theta}+\frac{\partial x_N^\gamma}{\partial\theta}=\left(\frac{-2n\frac{1}{b}-2n^2+\delta^2}{2\left(\frac{1}{b}+n\right)}\right)\frac{\partial p_G}{\partial\theta}+\left(\frac{2\delta\frac{1}{b}+\delta n}{2\left(\frac{1}{b}+n\right)}\right)\frac{\partial p_G}{\partial\theta}=\left(\frac{-2n\frac{1}{b}+2\delta\frac{1}{b}-2n^2+\delta^2+\delta n}{\left(\frac{2\delta\frac{1}{b}+\delta n}{2\left(\frac{1}{b}+n\right)}\right)\frac{\partial p_G}{\partial\theta}}\right)\frac{\partial p_G}{\partial\theta}<0$$

Food utilization: utility from food consumption.

Consumer surplus:

$$cs^\alpha=\int_0^{x_G^{\alpha*}}\left(a-bx_G^\alpha\right)dx_G^\alpha-p_G^*x_G^{\alpha*}=ax_G^\alpha-b\frac{\left(x_G^\alpha\right)^2}{2}-p_Gx_G^\alpha=\left(a-p_G\right)x_G^\alpha-\frac{b}{2}\left(x_G^\alpha\right)^2$$

$$cs^\beta=\int_0^{x_G^{\beta*}}\left(a-bx_N^\beta\right)dx_N^\beta-p_N^*x_N^{\beta*}=ax_N^\beta-b\frac{\left(x_N^\beta\right)^2}{2}-p_Nx_N^\beta=\left(a-p_N\right)x_N^\beta-\frac{b}{2}\left(x_N^\beta\right)^2$$

$$cs^\gamma=\int_0^{x_G^{\gamma*}}\left(a-bx_G^\gamma-hx_N^\gamma\right)dx_G^\gamma-p_G^*x_G^{\gamma*}+\int_0^{x_N^{\gamma*}}\left(a-hx_G^\gamma-bx_N^\gamma\right)dx_N^\gamma-p_N^*x_N^{\gamma*}$$

$$=\left(a-p_G\right)x_G^\gamma-\frac{b}{2}\left(x_G^\gamma\right)^2-2hx_G^\gamma x_N^\gamma+\left(a-p_N\right)x_N^\gamma-\frac{b}{2}\left(x_N^\gamma\right)^2$$

The marginal effects:

$$\frac{\partial cs^\alpha}{\partial\theta}=-\frac{\partial p_G}{\partial\theta}x_G^\alpha+\left(a-p_G\right)\frac{\partial x_G^\alpha}{\partial\theta}-bx_G^\alpha\frac{\partial x_G^\alpha}{\partial\theta}=-\frac{\partial p_G}{\partial\theta}x_G^\alpha+\left(a-p_G\right)\frac{\partial x_G^\alpha}{\partial\theta}+x_G^\alpha\frac{\partial p_G}{\partial\theta}$$

$$=-\frac{\partial p_G}{\partial\theta}x_G^\alpha+\left(a-p_G\right)\frac{\partial x_G^\alpha}{\partial\theta}+x_G^\alpha\frac{\partial p_G}{\partial\theta}=\underbrace{\left(a-p_G\right)}_{>0}\underbrace{\frac{\partial x_G^\alpha}{\partial\theta}}_{<0}<0$$

$$\frac{\partial cs^\beta}{\partial\theta}=-\frac{\partial p_N}{\partial\theta}x_N^\beta+\left(a-p_N\right)\frac{\partial x_N^\beta}{\partial\theta}-bx_N^\beta\frac{\partial x_N^\beta}{\partial\theta}=-\frac{\partial p_N}{\partial\theta}x_N^\beta+\left(a-p_N\right)\frac{\partial x_N^\beta}{\partial\theta}+x_N^\beta\frac{\partial p_N}{\partial\theta}=\left(a-p_N\right)\frac{\partial x_N^\beta}{\partial\theta}>0$$

$$\frac{\partial cs^\gamma}{\partial\theta}=-\frac{\partial p_G}{\partial\theta}x_G^\gamma+\left(a-p_G\right)\frac{\partial x_G^\gamma}{\partial\theta}-bx_G^\gamma\frac{\partial x_G^\gamma}{\partial\theta}-2hx_N^\gamma\frac{\partial x_G^\gamma}{\partial\theta}-2hx_G^\gamma\frac{\partial x_N^\gamma}{\partial\theta}-\frac{\partial p_N}{\partial\theta}x_N^\gamma+\left(a-p_N\right)\frac{\partial x_N^\gamma}{\partial\theta}-bx_N^\gamma\frac{\partial x_N^\gamma}{\partial\theta}$$

$$=\underbrace{\left(a-p_G\right)\frac{\partial x_G^\gamma}{\partial\theta}}_{<0}\underbrace{-2hx_N^\gamma\frac{\partial x_G^\gamma}{\partial\theta}}_{>0}\underbrace{-2hx_G^\gamma\frac{\partial x_N^\gamma}{\partial\theta}}_{<0}\underbrace{+\left(a-p_N\right)\frac{\partial x_N^\gamma}{\partial\theta}}_{>0}\underbrace{-\frac{\partial p_G}{\partial\theta}x_G^\gamma-\frac{\partial p_N}{\partial\theta}x_N^\gamma}_{<0}\underbrace{-bx_G^\gamma\frac{\partial x_G^\gamma}{\partial\theta}}_{>0}\underbrace{-bx_N^\gamma\frac{\partial x_N^\gamma}{\partial\theta}}_{<0}$$

It is ambiguous.
